# Supplementary material for: Clinical ultrasound, photoacoustic, and fluorescence image-guided lymphovenous anastomosis microsurgery via a transparent ultrasound transducer array
Source: Nat Commun. 2025 Nov 7;16:9853. doi: 10.1038/s41467-025-64827-8 (PMC12594831; doi:10.1038/s41467-025-64827-8)
Supplement: Supplementary file 1 — Supplementary Information File [file 41467_2025_64827_MOESM1_ESM.pdf]

Title: Clinical Ultrasound, Photoacoustic, and Fluorescence Image-guided Lymphovenous Anastomosis Microsurgery via a Transparent Ultrasound Transducer Array

Authors

Jeongwoo Park[1,2,†]
Donghyeon Oh[1,†]
Jinhee Yoo[1,†]
Honghyeon Ha[1,3]
Donggyu Kim[1]
Hyung Ham Kim[1,*]
Yujin Myung[4,*]
Chulhong Kim[1,5,*]

Affiliations

[1]Department of Electrical Engineering, Convergence IT Engineering, Mechanical Engineering, Medical Science and Engineering and Medical Device Innovation Center, Pohang University of Science and Technology (POSTECH), Pohang 37673, Republic of Korea
[2]Department of Biomedical Convergence Science and Technology, Advanced Bioconvergence, and Cell and Matrix Research Institute, Kyungpook National University, Daegu, 41566, Republic of Korea
[3]SonicLab Inc., Siheung, Republic of Korea
[4]Department of Plastic and Reconstructive Surgery, Seoul National University Bundang Hospital, Seoul National University College of Medicine, Seongnam 13620, Republic of Korea
[5]Opticho Inc., Pohang, Republic of Korea

[†]These authors have contributed equally
[*]Corresponding authors

Chulhong Kim
Email: chulhong@postech.edu

Yujin Myung
Email: surgene@snu.ac.kr

Hyung Ham Kim
Email: david.kim@postech.ac.kr

This PDF file includes the following contents:

Figure S1: Capacitances of each element in the TUT-array before and after high-voltage poling

Figure S2: Krimholtz–Leedom–Matthaei (KLM) model–based simulation results

Figure S3: Acoustic beam profiles of the TUT-array

Figure S4: Schematic of the triple-modal USI/PAI/FLI imaging system with an opto-US imaging probe

Figure S5: Timing sequence of PAI, USI, and FLI signal acquisition

Figure S6: Performance evaluation of USI by the opto-US probe

Figure S7: Performance evaluation of PAI by the opto-US probe

Figure S8: Comparison of US and PA B-scan images acquired using the TUT-array with coaxial versus oblique laser illumination.

Figure S9: Performance evaluation of FLI by the opto-US probe

Figure S10: Photograph of the *in vitro* experimental setup for multimodal USI/PAI/FLI using the opto-US probe

Figure S11: *In vivo* multispectral PAI of an indocyanine green (ICG)-injected rat and quantification of PA signals in lymph vessels

Figure S12: Triple-modal USI/PAI/FLI guided microsurgical lymphaticovenous anastomosis (LVA) of Patient 2.

Figure S13: An optical fluence Monte Carlo simulation in biological tissue highlights deep optical penetration with coplanar illumination.

Figure S14: Comparison of PAI with coaxial and oblique laser alignments

Table S1: Imaging parameters, acquisition time, and post-processing time for each experimental condition.

Supplementary Figure 1

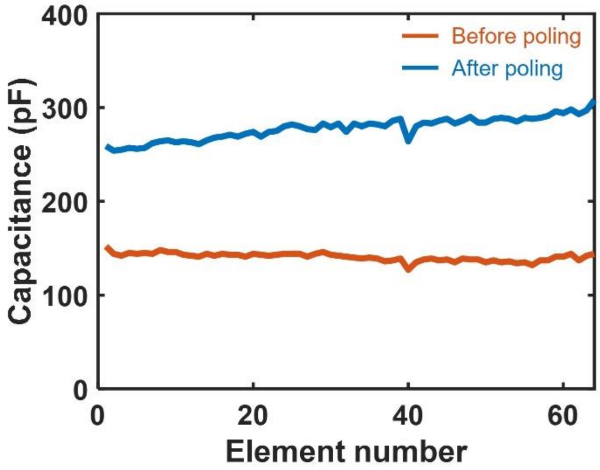

Figure S1. Comparison of the capacitance of each element in the TUT-array before and after the high-voltage poling process. All elements exhibited a significant increase in capacitance following the poling process.

Supplementary Figure 2

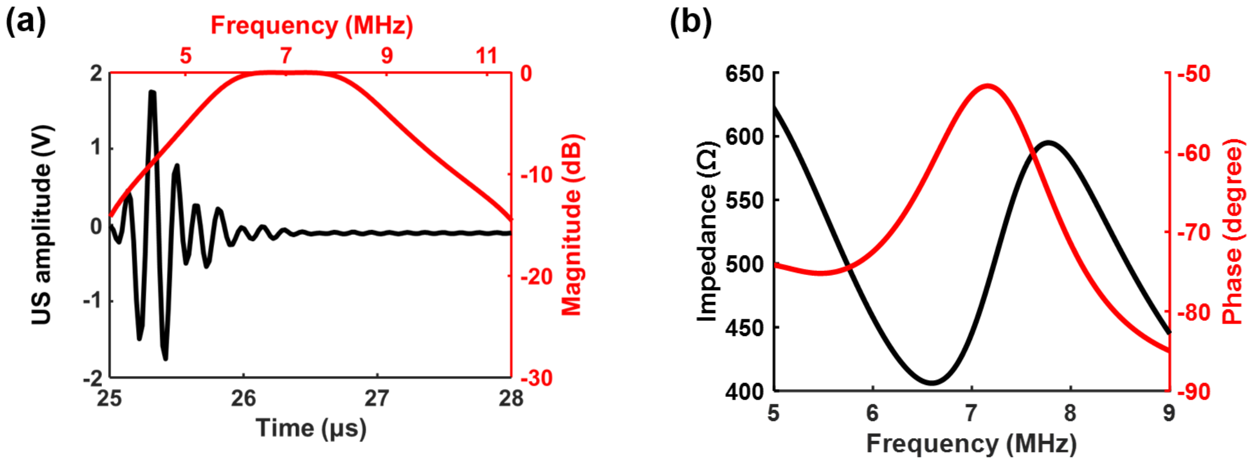

Figure S2. Krimholtz-Leedom-Matthaei (KLM) model-based simulation results: (a) pulse-echo response, where the black line represents the time-domain waveform and the red line represents the frequency spectrum; and (b) electrical impedance, where the black line denotes the impedance magnitude and the red line denotes the phase.

Supplementary Figure 3

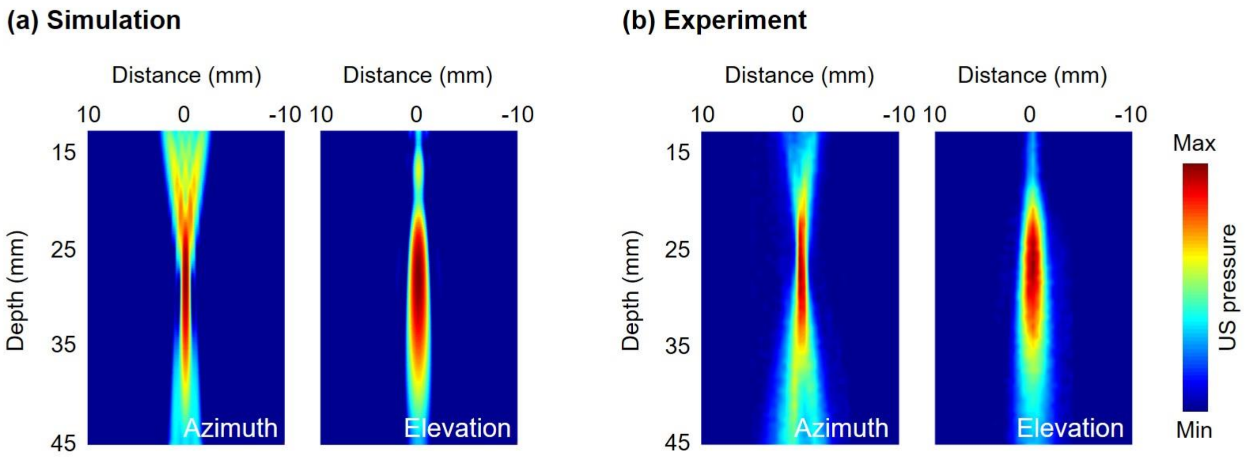

Figure S3. Acoustic beam profiles of the TUT-array. (a) Field-II simulation results and (b) experimental results measured using a hydrophone. Both simulation and experiment demonstrate comparable focal patterns along the azimuth and elevation axes.

Supplementary Figure 4

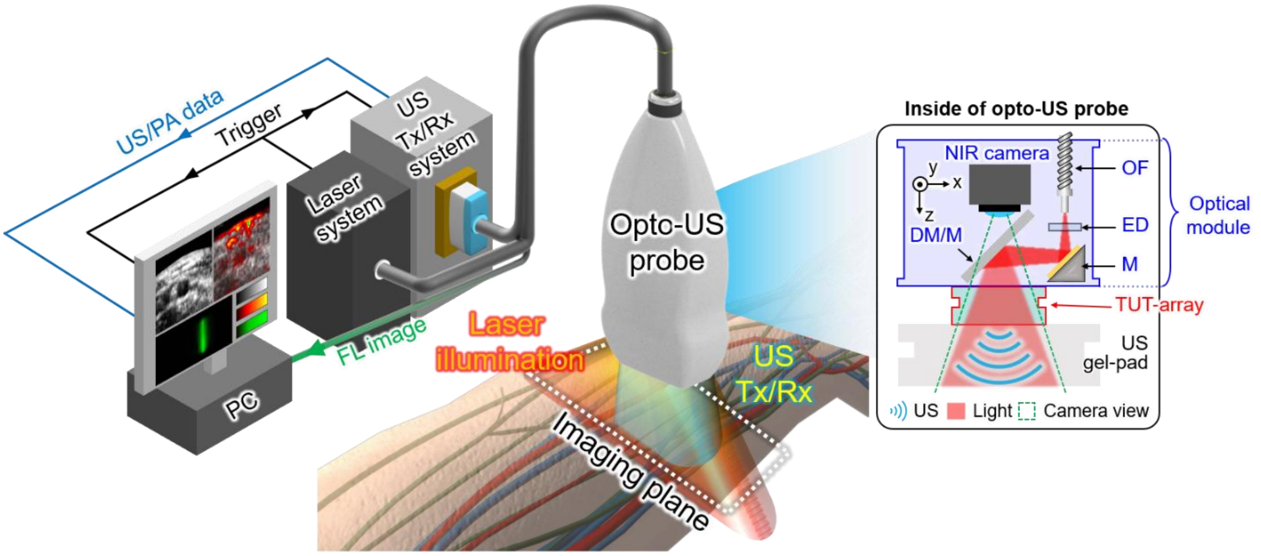

Figure S4. Schematic of the triple-modal USI/PAI/FLI imaging system with an opto-US imaging probe. The US Tx/Rx system connects to the TUT-array, while the laser system delivers light through an optical fiber (OF). The trigger signal from the laser system enables the US Tx/Rx system to be synchronized with the PC and transfers the acquired US and PA data to the PC. An NIR camera is connected to the PC via USB and transmits FL images directly to the PC. DM, dichroic mirror; M, mirror; ED, engineered diffuser; and OF, optical fiber.

Supplementary Figure 5

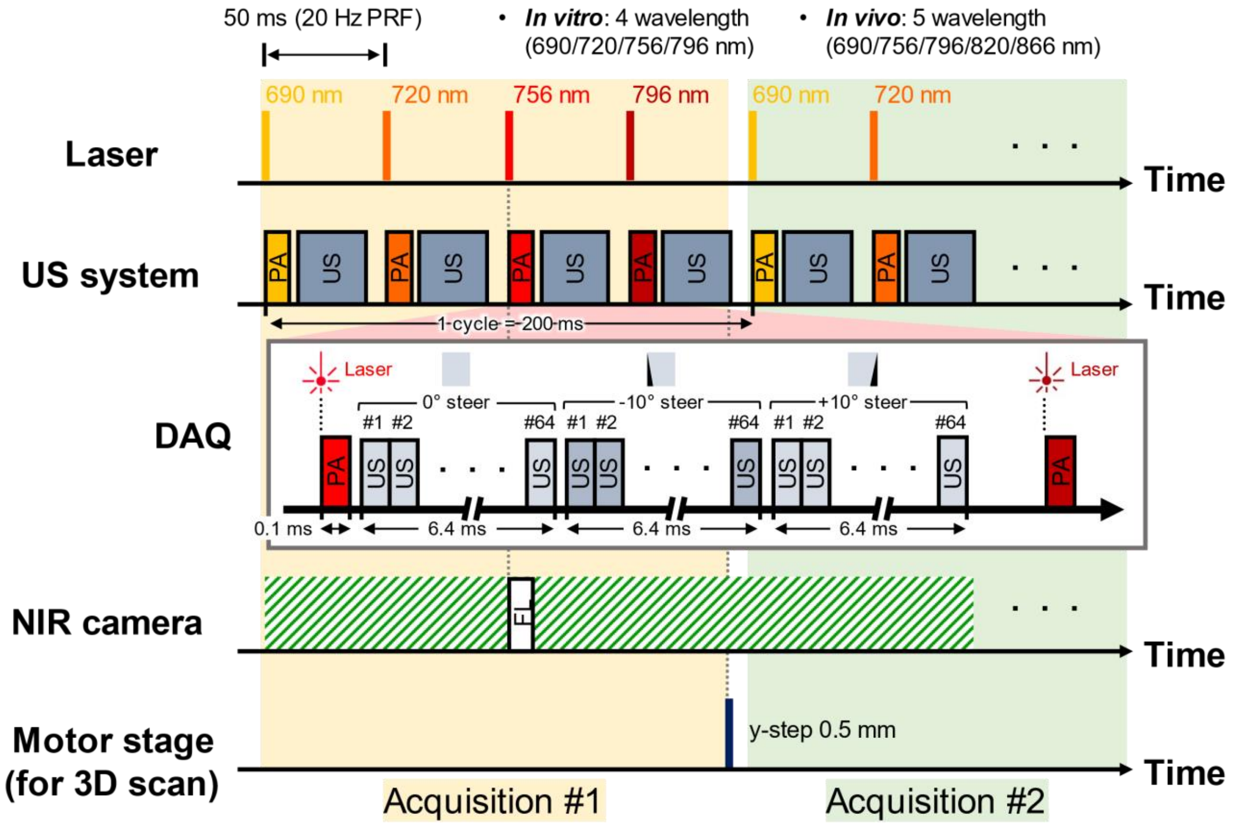

Figure S5. Timing sequence of PAI, USI, and FLI signal acquisition. The illustration assumes multispectral PAI at four wavelengths, with the number of wavelengths adjusted depending on the experimental settings. The outward trigger was optionally given to the external motor stage for 3D scanning.

Supplementary Figure 6

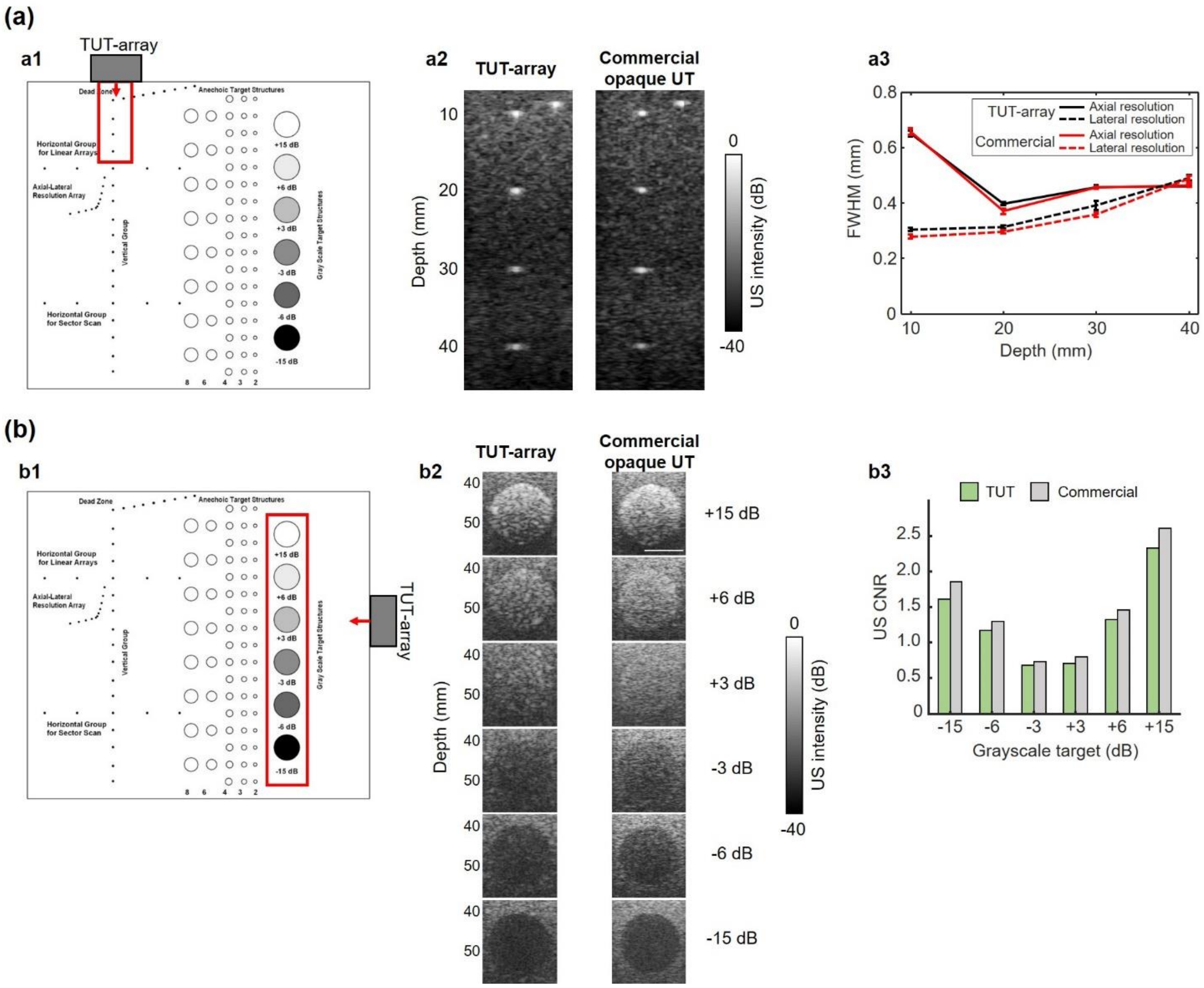

Figure S6. Performance evaluation of USI by the opto-US probe. (a) Spatial resolution comparison: (a1) imaging region of line targets in a standard US phantom, (a2) US B-scan images obtained from the TUT-array and a commercial opaque ultrasound transducer (UT), (a3) measured spatial resolutions of the TUT-array and the commercial opaque UT (mean ± standard error, n = 3, independent experiments). (b) Comparison of US contrast-to-noise ratios (CNRs): (b1) imaging region of grayscale targets in the standard US phantom, (b2) US B-scan images from the TUT-array and the commercial opaque UT, (b3) measured US CNRs for both the TUT-array and commercial opaque UT.

Supplementary Figure 7

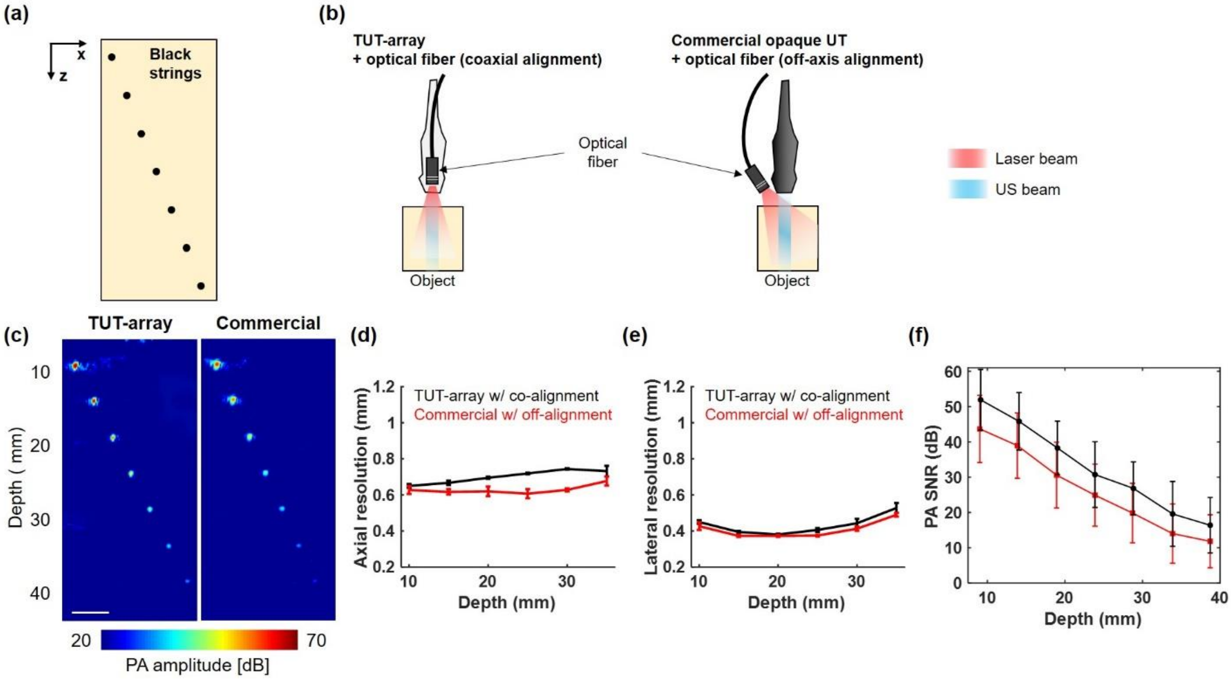

Figure S7. Performance evaluation of PAI by the opto-US probe. (a) Cross-section of black strings in a tissue-mimicking phantom. (b) Schematic comparison of the TUT-array with an optical fiber in coaxial alignment and a commercial opaque UT with an optical fiber in oblique alignment. (c) Comparison of PA B-scan images between the TUT-array with coaxial alignment and the commercial UT with oblique alignment. Comparison of the (d) axial and (e) lateral PAI resolutions, and (f) PA SNR between the TUT-array and the commercial UT (mean ± standard error, n = 3, independent experiments).

Supplementary Figure 8

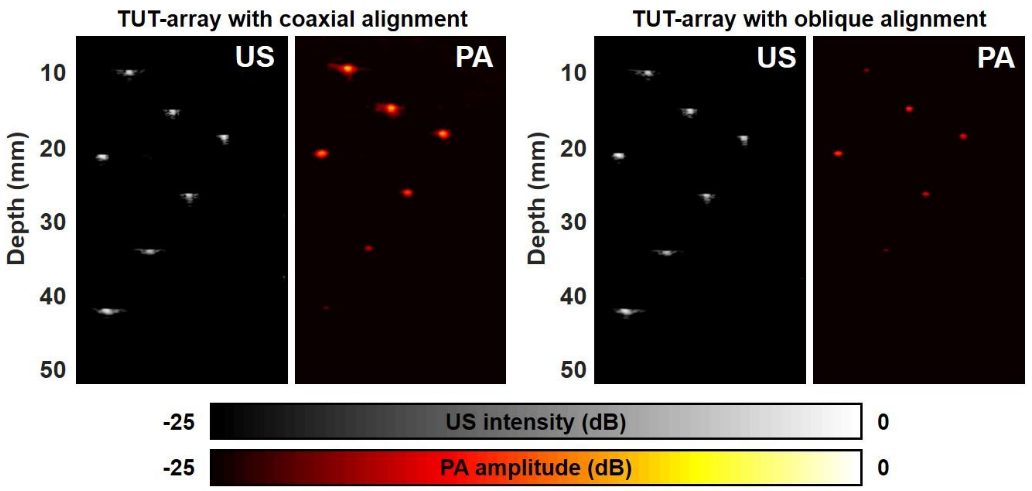

Figure S8. Comparison of US and PA B-scan images acquired using the TUT-array with coaxial versus oblique laser illumination.

Supplementary Figure 9

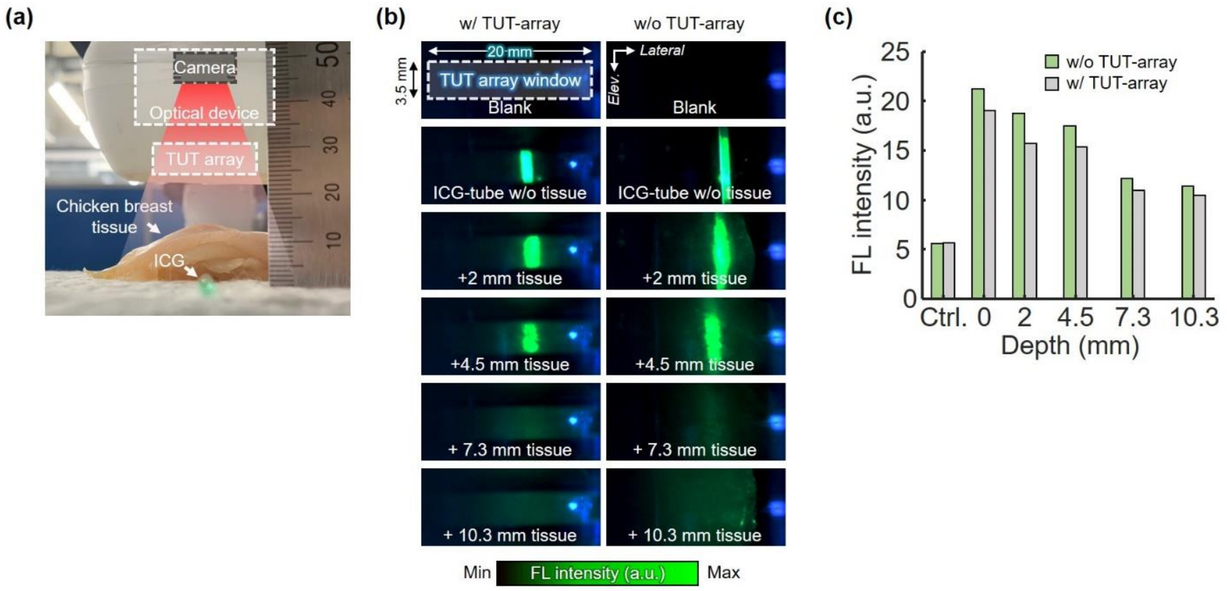

Figure S9. Performance evaluation of FLI by the opto-US probe. (a) Photograph of the experimental setup. (b) Comparison of FL SNRs with and without the TUT-array.

Supplementary Figure 10

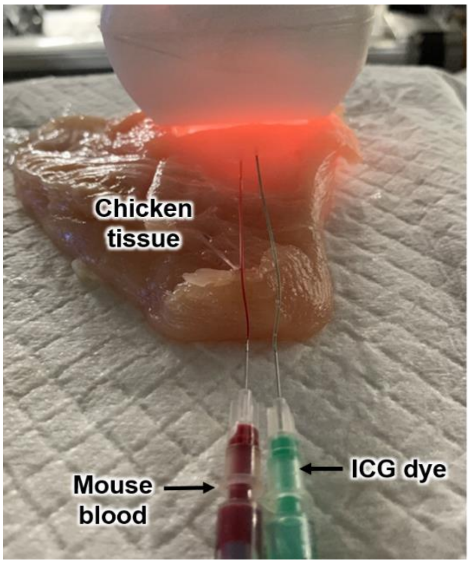

Figure S10. Photograph of the in vitro experimental setup for multimodal USI/PAI/FLI using the opto-US probe.

Supplementary Figure 11

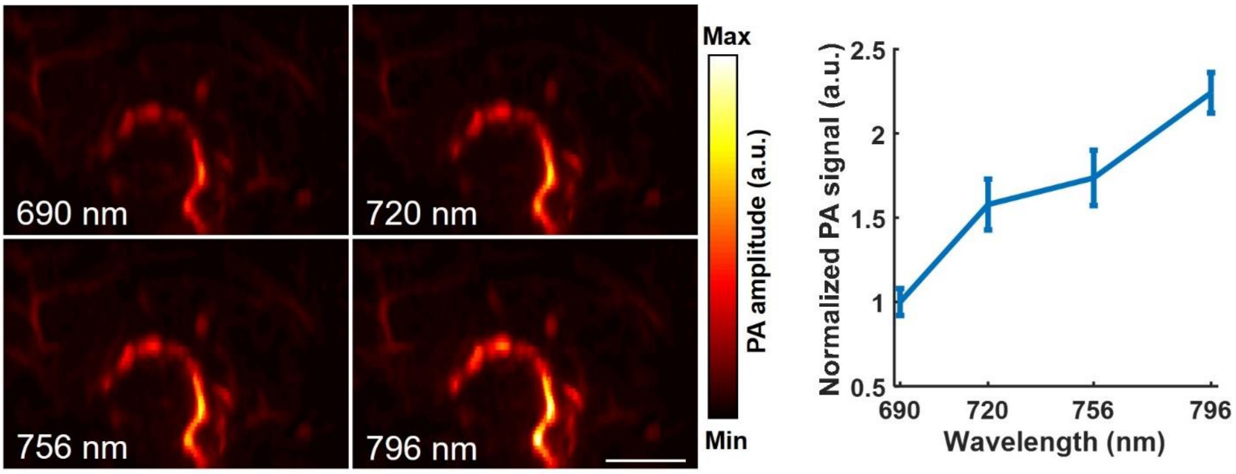

Figure S11. *In vivo* multispectral PAI of an indocyanine green (ICG)-injected rat and quantification of PA signals in lymph vessels (mean ± standard error, n = 3, independent experiments). Scale bar = 1 cm.

Supplementary Figure 12

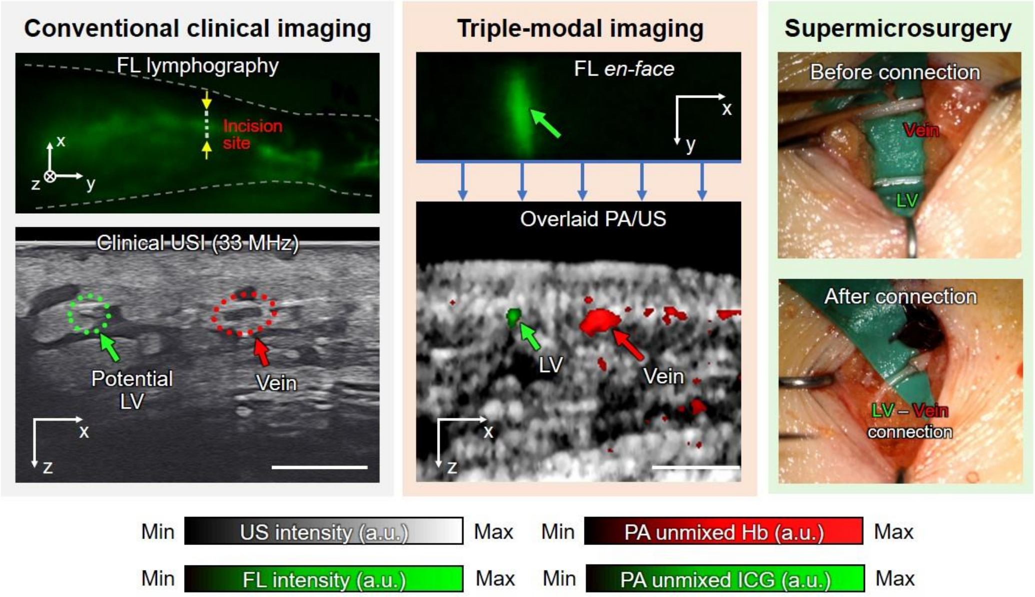

Figure S12. Triple-modal USI/PAI/FLI guided microsurgical lymphaticovenous anastomosis (LVA) of Patient 2. Conventional clinical imaging examination. Triple-modal USI/PAI/FLI examination of lymphatic vessels (LVs) and blood vessels. Photographs acquired before and after LVA supermicrosurgery. Scale bar = 5 mm. All values are expressed in a.u., which denotes arbitrary units.

Supplementary Figure 13

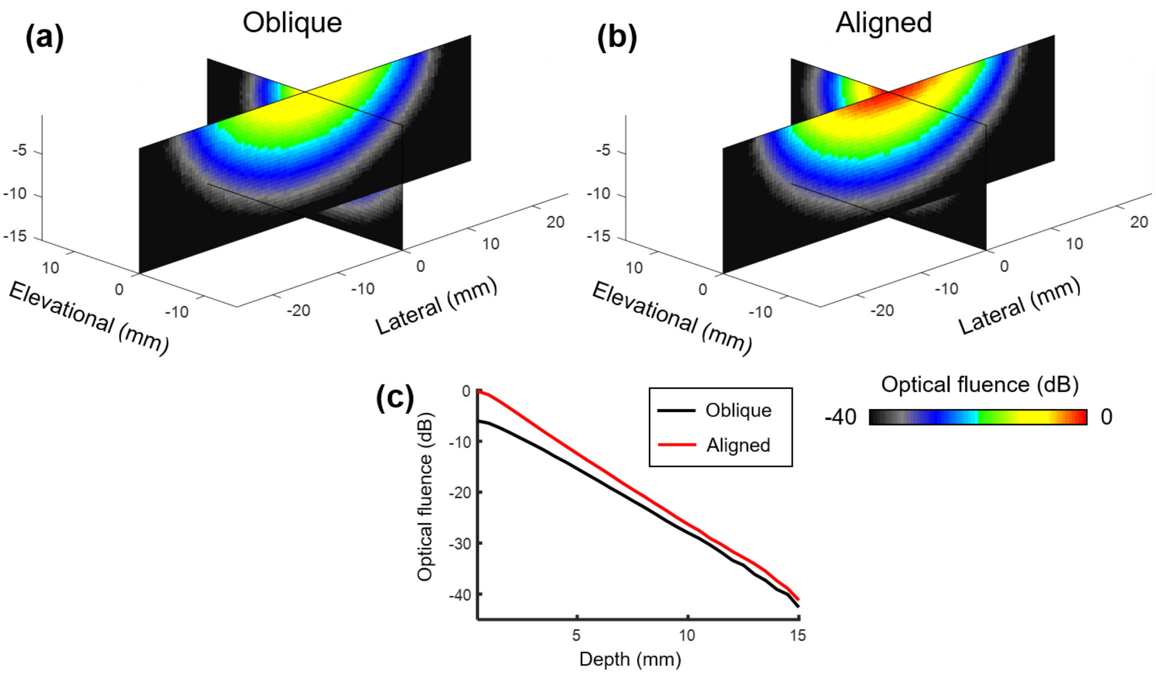

Figure S13. An optical fluence Monte Carlo simulation in biological tissue highlights deep optical penetration with coplanar illumination. 3D normalized optical fluence represented in the x-z acoustic plane (y=-0 mm) and y-z central plane (x=0) from (a) conventional oblique illumination and (b) coplanar illumination. (c) Optical fluence variation with depth at the central axis (x=0, y=0).

Supplementary Figure 14

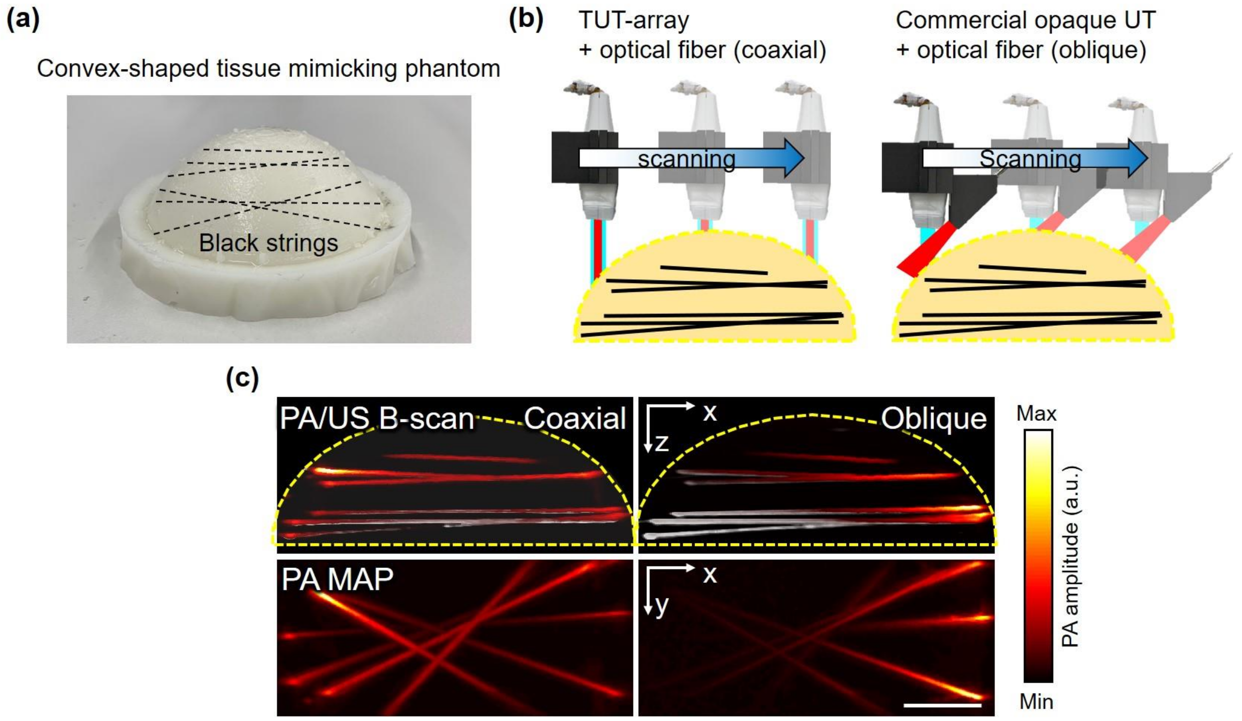

Figure S14. Comparison of PAI with coaxial and oblique laser alignments. (a) Photograph of a convex tissue-mimicking phantom. (b) Experimental schematic showing coaxial laser alignment with the TUT-array and oblique laser alignment with a commercial opaque UT. (c) PA/US overlaid B-scan images and PA maximum amplitude projection (MAP) images for each laser alignment configuration. Scale bar = 1 cm. All values are expressed in a.u., which denotes arbitrary units.

Supplementary Table S1

Table S1. Imaging parameters, acquisition time, and post-processing time for each experimental condition.

	Animal	Human arm	Lymphedema patients
**Imaging Modality**	PA/US/FL	PA/US	PA/US/FL
**Dimension**	3D	3D	2D
**Scanning strategy**	Motor scanning	Motor scanning	Handheld
**Field-of-view (mm)**	45×30×40 mm^3 (Mosaic; X-, Y-, and Z-axis)	30×50×40 mm^3 (Mosaic; X-, Y-, and Z-axis)	20×40 mm^3 (X-, and Z-axis)
**2D scan frame rate**			
PA - single wavelength	20 Hz	20 Hz	20 Hz
Number of Wavelengths	4	5	5
PA – multispectral	5 Hz	4 Hz	4 Hz
US	20 Hz	20 Hz	20 Hz
Interleaved multispectral PA+US	5 Hz	4 Hz	5 Hz
FL camera	60 Hz (Live)	-	60 Hz (Live)
Number of frames per location	1	1	25
**3D scan**			
Elevational step	0.5 mm	0.5 mm	-
Number of Y-slices	60	100	1
Number of mosaic batches	4	2	-
**Total acquisition time (acquisition, reconstruct & save)**	8 min 48 sec	6 min 50 sec	30 sec
