# Supplementary material for: Clinical ultrasound, photoacoustic, and fluorescence image-guided lymphovenous anastomosis microsurgery via a transparent ultrasound transducer array
Source: Nat Commun. 2025 Nov 7;16:9853. doi: 10.1038/s41467-025-64827-8 (PMC12594831; doi:10.1038/s41467-025-64827-8)
Supplement: Supplementary file 2 — Description of Additional Supplementary Files [file 41467_2025_64827_MOESM2_ESM.pdf]

Description of Additional Supplementary Files

Supplementary Movie 1: 3D triple-modal (ultrasound, photoacoustic, and fluorescence) mapping of blood and lymphatic vessels in live animals using the handheld opto-US probe

Supplementary Movie 2: 3D Clinical 3D functional photoacoustic/ultrasound (PA/US) vascular imaging of the human arm using the handheld opto-US probe

Supplementary Movie 3: Simultaneous photoacoustic/ultrasound (PA/US) imaging of cardiac circulation dynamics

Supplementary Movie 4: Real-time triple-modal (ultrasound, photoacoustic, and fluorescence) imaging of the ICG-infused forearm of a healthy volunteer using the handheld opto-US probe

Supplementary Movie 5: Preoperative real-time triple-modal (ultrasound, photoacoustic, and fluorescence) imaging of the leg of a lymphedema patient using the handheld opto-US probe.
